# Cell cycle block by p53 activation reduces SARS-CoV-2 release in infected alveolar basal epithelial A549-hACE2 cells

**DOI:** 10.3389/fphar.2022.1018761

**Published:** 2022-12-13

**Authors:** Giada Lodi, Valentina Gentili, Fabio Casciano, Arianna Romani, Giorgio Zauli, Paola Secchiero, Enrico Zauli, Carolina Simioni, Silvia Beltrami, Mercedes Fernandez, Roberta Rizzo, Rebecca Voltan

**Affiliations:** ^1^ Department of Environmental and Prevention Sciences and LTTA Centre, University of Ferrara, Ferrara, Italy; ^2^ Department of Chemical, Pharmaceutical and Agricultural Sciences, University of Ferrara, Ferrara, Italy; ^3^ Department of Translational Medicine and LTTA Centre, University of Ferrara, Ferrara, Italy; ^4^ Interdepartmental Research Center for the Study of Multiple Sclerosis and Inflammatory and Degenerative Diseases of the Nervous System, University of Ferrara, Ferrara, Italy; ^5^ Research Department, King Khaled Eye Specialistic Hospital, Riyadh, Saudi Arabia; ^6^ Department of Translational Medicine, University of Ferrara, Ferrara, Italy; ^7^ Department of Life Sciences and Biotechnology, University of Ferrara, Ferrara, Italy

**Keywords:** nutlin-3, MDM2, SARS-CoV-2, cell cycle, IL-6, p53, NF-kB

## Abstract

SARS-CoV viruses have been shown to downregulate cellular events that control antiviral defenses. They adopt several strategies to silence p53, key molecule for cell homeostasis and immune control, indicating that p53 has a central role in controlling their proliferation in the host. Specific actions are the stabilization of its inhibitor, MDM2, and the interference with its transcriptional activity. The aim of our work was to evaluate a new approach against SARS-CoV-2 by using MDM2 inhibitors to raise p53 levels and activate p53-dependent pathways, therefore leading to cell cycle inhibition. Experimental setting was performed in the alveolar basal epithelial cell line A549-hACE2, expressing high level of ACE2 receptor, to allow virus entry, as well as p53 wild-type. Cells were treated with several concentrations of Nutlin-3 or RG-7112, two known MDM2 inhibitors, for the instauration of a cell cycle block steady-state condition before and during SARS-CoV-2 infection, and for the evaluation of p53 activation and impact on virus release and related innate immune events. The results indicated an efficient cell cycle block with inhibition of the virion release and a significant inhibition of IL-6, NF-kB and IFN-λ expression. These data suggest that p53 is an efficient target for new therapies against the virus and that MDM2 inhibitors deserve to be further investigated in this field.

## 1 Introduction

The two last decades have shown great sanitary emergencies due to the pandemic diffusion of new human-infecting betacoronaviruses, in particular the highly pathogenic severe acute respiratory syndrome coronavirus 1, SARS-CoV-1, the middle east respiratory syndrome-related coronavirus, MERS-CoV, and more recently the severe acute respiratory syndrome coronavirus 2, SARS-CoV-2 (de Wit et al., 2016; Zhu et al., 2020; Lu et al., 2020). The attention of the scientific literature towards the coronavirus disease COVID-19 pandemic has clarified many aspects of infection on target cells, affirming the key role of the angiotensin converting enzyme 2 (ACE2) receptor and the transmembrane protease serine 2 (TMPRSS2) co-receptor in tropism and pathogenesis (Sajuthi et al., 2020; Zipeto et al., 2020). Currently, many of the intracellular mechanisms adopted by the virus to force the infected cell to produce new virions are known (Jackson et al., 2022), including cell cycle control to slow down normal cellular activities diverting resources towards the transcription of the viral genomes and the translation of its own proteins, while other mechanisms are still to be understood. Moreover, the virus can disarm the cell from its defense mechanisms capable of counteracting viral infection, such as programmed cell death, leading to a severe inflammatory status, with pro-inflammatory cytokine release and reduced interferon response, which is the typical fingerprint of an uncontrolled spread of the virus into the body (Hu et al., 2021).

One of the targets of the virus that plays an important role both in the mechanisms of innate immunity and in the control of the cell cycle and other pathways that regulate cell replication, damage repair, apoptosis and metabolism is the p53 protein (Cardozo and Hainaut, 2021; Ma-Lauer et al., 2016; Zauli et al., 2020; Harford et al., 2022; Kumar et al., 2022; Zauli et al., 2022). Indeed, the tumor suppressor p53 is a pleiotropic protein controlling cell homeostasis and cell response to stress events, deciding cell fate. In healthy conditions, p53 is kept to a low threshold level through the interaction with Murine Double Minute-2 (MDM2), that post-transcriptionally controls p53 with ubiquitination and addresses it to proteasome. When a stress signal activates p53, the protein can direct differential intracellular mechanisms, depending on the cellular and tissue context and on the type, strength and duration of insult, by transcriptional regulation of target genes or by protein-protein interactions (Kastenhuber and Lowe, 2017). In response to viruses, p53 has a recognized role in contrasting the infection principally through cell death induction as a part of the antiviral innate immunity, together with the regulation of cytokine production and nuclear factor kappa light chain enhancer of activated B cells, NF-kB signaling (Muñoz-Fontela et al., 2016; Milani et al., 2022).

SARS-CoV, as observed for other viruses, have evolved specific molecular mechanisms to contrast p53, in order to avoid the host response to infection. Indeed, the viral Papain-Like Proteases (PLPs) act directly on MDM2 and on RING finger and CHY zinc finger domain-containing protein 1 (RCHY1) the “core” ubiquitin ligases involved in p53 ubiquitination, promoting their stabilization, and forwarding p53 to the proteasome degradation (Ma-Lauer et al., 2016; Yuan et al., 2015). Moreover, viral Non-Structural Protein 5 (NSP5) can interfere with p53 transcriptional activity, impacting the regulation of its target genes (Kumar et al., 2022). Vice versa, overexpression of recombinant *TP53* gene has shown some capacity to moderate SARS-CoV replication (Ma-Lauer et al., 2016; Kumar et al., 2022).

Following all these evidence and considerations, we hypothesize that the pharmacological use of MDM2 inhibitors for the non-genotoxic upregulation of p53 could be effective to contrast SARS-CoV-2 infection. To this purpose, we settled the work using a cellular model of alveolar basal epithelium with wild-type *TP53* status and permissive to the SARS-CoV-2 infection thanks to the hyper-expression of ACE2 receptor, the cell line A549-hACE2. In this model, we assessed the effects of Nutlin-3, the first cis-imidazoline small molecule designed to inhibit MDM2, and RG-7112, its most innovative and powerful derived version (Vassilev et al., 2004; Tovar et al., 2013). The two molecules act on MDM2 in a similar way, entering to the p53 binding pocket of MDM2, but possess different solubility and inhibition capacity (IC_50_ of 18 nM for RG-7112 and 90 nM for Nutlin-3, as calculated in cell-free assays [https://www.selleckchem.com/search.html?sp=MDM2%252520inhibitor];). The aim of the work was to investigate a new therapeutical approach of MDM2 inhibitors for the control of SARS-CoV-2 infection through cell cycle inhibition and innate immune control.

## 2 Materials and methods

### 2.1 Cell cultures and treatments with MDM2 inhibitors

A549-hACE2 cells were a kind gift of Professor Arnaldo Caruso and Professor Francesca Caccuri (University of Brescia, Italy) and were grown in Dulbecco’s Modified Eagle’s Medium (DMEM, Carlo Erba, Cornaredo, IT) supplemented with 10% fetal bovine serum (FBS, Gibco, Invitrogen Corporation, NY, United States) and 1% Penicillin-Streptomycin-Glutamine 100X (Sigma-Aldrich, Darmstadt, Germany). Cells were maintained at 37°C in a humidified atmosphere with 5% CO_2_ and trypsinized every 2-3 days for passages or experiments. Cultures were routinely checked for *mycoplasma* contamination.

The day before the experiments, cells were seeded at a density of 8 × 10^4^ cells/mL/well in 12 well plates. The following day, cells were treated with Nutlin-3 (Cayman Chemicals, Michigan, United States) or RG-7112 (Selleckchem, Planegg, Germany) at the predetermined concentrations of 0.1, 1, and 2.5 µM. Cells left with complete medium (untreated, Unt) or treated with dimethyl sulfoxide (DMSO) as drug vehicle were used as internal controls. After 24 h of treatment, cells were harvested for cell cycle and cell growth, apoptosis and western blotting analyses, or treatments were replaced in complete medium supplemented with 5% fetal bovine serum and cells were incubated for 48 h (reaching 72 h of treatment) and then harvested for analyses. Some cultures were infected with SARS-CoV-2 as described in the following paragraphs. The experimental workflow is represented in Supplementary Figure S1.

### 2.2 Cell viability and cell cycle profile

Cells treated for 24 or 72 h with Nutlin-3 or RG-7112 (0.1, 1 and 2.5 µM) and relative controls were analyzed for cell viability by Trypan blue dye exclusion. At the same time points, cell cycle profile and hypodiploid cell percentage were examined by 5-bromodeoxyuridine (BrdU; Sigma, St Louis, MO, United States) incorporation, as previously reported (Romani et al., 2021). Briefly, after treatment, cells were incubated with 10 µM BrdU (Sigma- Aldrich, Darmstadt, Germany) for 1 hour at 37°C, trypsinized and collected together with the floating cells harvested with the medium before trypsin treatment. Total cells were then stained with anti-BrdU primary antibody (BD Pharmingen TM, San Diego, CA, United States), goat F (ab’)2 anti-mouse IgG (H + L) fluorescein isothiocyanate-conjugated secondary antibody (Beckman Coulter, Brea, CA) and propidium iodide (PI; Sigma, St Louis, MO, United States). Cells were acquired using the FACS Calibur flow cytometer (BD Bioscience, San Josè, CA, United States), and data were analyzed using FlowJo software (Tree Star, Ashland, OR, United States).

### 2.3 SARS-CoV-2 propagation and infection of A549-hACE2 cells

SARS-CoV-2 inoculum, originally isolated from a patient with COVID-19 (genome sequences available at GenBank (SARS-CoV-2-UNIBS-AP66: ERR4145453) was a kind gift of Professor Arnaldo Caruso. SARS-CoV-2 was propagated in Vero E6 cells in Modified Eagle Medium (MEM; Gibco, Waltham, MA) supplemented with 2% FBS, and titrated by plaque assay as described previously (Caccuri et al., 2022). A549-hACE2 cells were grown to 80% confluency and treated with Nutlin-3 or RG-7112 for 24 h before infection. Then, the cells were infected at a multiplicity of infection (MOI) of 0.01, and incubated for 1 h at 37°C. After adsorption, viral inoculum was removed, cells were washed twice with phosphate-buffered saline (PBS) and treatments were restored in fresh medium supplemented with 2% FBS. Samples were collected for analyses 48 h post-infection.

All the experiments with SARS-CoV-2 were performed in a biosafety level-3 (BSL-3) laboratory.

### 2.4 SARS-CoV-2 quantification and gene expression analysis by real time-PCR

Viral genomes were quantitated by real time PCR both in cellular supernatants and intracellularly. Total RNA was extracted from clarified cell culture supernatants using PureLink Viral RNA/DNA Mini Kit (Invitrogen, Waltham, MA, United States), and from cells using PureLink RNA Mini Kit (Invitrogen) according to the manufacturer’s instructions. RNA was reverse-transcripted with SuperScript IV VILO (Invitrogen). SARS-CoV-2 quantification in supernatants was performed using the PowerUp SYBR Green Master Mix (Thermo Fisher, Milan, Italy) as follows: RBD-qF1: 5′-CAA​TGG​TTT​AAC​AGG​CAC​AGG-3′ and RBD-qR1: 5′-CTC​AAG​TGT​CTG​TGG​ATC​ACG-3′ (Gentili et al., 2021). Intracellular SARS-CoV-2 was quantified using TaqMan universal II Master Mix (Thermo Fisher, Milan, Italy) as follows: Sp-F: 5′-CCA​GAT​CCA​TCA​AAA​CCA​AGC-3’; Sp-R: 5′-TGC​ACA​AAT​GAG​GTC​TCT​AGC-3’; Sp-P: 5′-FAM-AGTGACACGCAGATGCTGGCT-BkFQ-3’. RNaseP assay was used as internal normalization control. For absolute quantification, dsDNA fragment (gBlock®, Integrated DNA Technologies, Coralville, IA, United States) based on the RBD sequence was used to generate a standard curve.

For transcript analysis, total RNA was extracted from cells using PureLink RNA Mini Kit (Invitrogen, Waltham, MA, United States), spectrophotometrically quantified, digested with DNAseI, and finally reverse transcribed. The obtained cDNA was amplified using PowerUp SYBR Green Master Mix (Thermo Fisher, Milan, Italy) with the following primer sets: glyceraldehyde-3-phosphate dehydrogenase, GAPDH, (Hs.PT.58.25887499. g), NF-kB (Hs.PT.58.20344216), interleukin 6, IL-6 (IL-6-F: TAG​GAC​TGG​AGA​TGT​CTG​AGG​CT IL-6-R: GAC​CGA​AGG​CGC​TTG​TGG​A), interferon lambda 1, IFN-λ1, (Hs.PT.56a.21113836. g). Relative quantification of mRNA levels for the samples was conducted using the 2^−ΔΔCT^, 2 (Delta Delta CT) method (Livak and Schmittgen, 2001), normalized to the constitutively expressed housekeeping gene GAPDH. For all experiments at least three replicates were performed.

### 2.5 Western blotting

After 24 and 72 h of treatment with Nutlin-3 or RG-7112 (0.1, 1 and 2.5 µM) or controls, cells were harvested for protein expression analysis by Western blotting, as previously described (Rimondi et al., 2021). Cells were lysed in buffer containing 50 mM Tris-Cl, pH 7.5, 150 mM NaCl, 0.1% sodium dodecyl phosphate 10%, 1% NP40, 0.25% sodium deossicolate and protease and phosphatase inhibitor mini tablets, EDTA-Free (1 tablet/10ml; Pierce™, Thermo Fisher Scientific, Massachusetts, United States). Protein concentration of extracts was determined by the bicinchoninic acid BCA Protein Assay Kit (Pierce™, Thermo Fisher Scientific, Massachusetts, United States). For Western blotting analysis, equal amounts of protein were separated on 10% SDS-PAGE and transferred into nitrocellulose membrane (Amersham, Merk, Darmstadt, Germany). Each gel was loaded with an appropriate internal control expressing the protein of interest and with a specific molecular weight marker.

Blots were incubated with the following antibodies: anti-p53; anti-Mdm2; (all purchased from Santa Cruz Biotechnology, Santa Cruz, CA), anti-human retinoblastoma protein clone G3-245 (anti-Rb, BD Pharminger, CA, United States) and anti-β-tubulin (Sigma-Aldrich, Darmstadt, Germany). After incubation with secondary antibody (anti-mouse IgG horseradish peroxidase HRP-conjugated; Sigma-Aldrich), immunoreactive proteins bands were detected with the enhanced chemiluminescent (ECL) Lightning kit (Perkin Elmer, Waltham, MA, United States). Acquisition and analysis were performed by Image QuantTM LASS 4000 imager and TL software (GE healthcare Bio-Sciences, Chalfont Saint Giles, Buckinghamshire, United Kingdom).

### 2.6 Statistical analysis

Statistical analysis of data was performed by GraphPad Prism version 9 software (GraphPad Software, San Diego, La Jolla, CA, United States). Data obtained from at least three independent experiments were tested for normal distribution by Shapiro-Wilks. The results were evaluated by paired Student’s t-test (for relative expression of target genes), or analysis of variance (ANOVA) followed by Bonferroni *post hoc* test for multiple corrections. Results were expressed as mean ± standard error of the mean (SEM), and significance was defined as *p* < 0.05.

## 3 Results

### 3.1 MDM2 antagonists are effective in inducing cell cycle block of alveolar basal epithelial A549-hACE2 cells

To verify the efficiency of treatments with the MDM2 inhibitors Nutlin-3 and RG-7112, we first investigated their biological effect on the cell cycle of A549-hACE2 cells, using a predetermined range of concentrations in order to avoid significant cytotoxic effects that could alter the next steps of the experimental design (Supplementary Figure S1). We have chosen the time points of 24 and 72 h post treatment to follow the experimental schedule dictated by the method of SARS-CoV-2 infection. The 24 h time point is needed to verify that at the time of inoculation with the virus the cells are already under the influence of the biological effect of the molecules under examination. The 72 h time point corresponds to 48 h post-infection.

**FIGURE 1:**
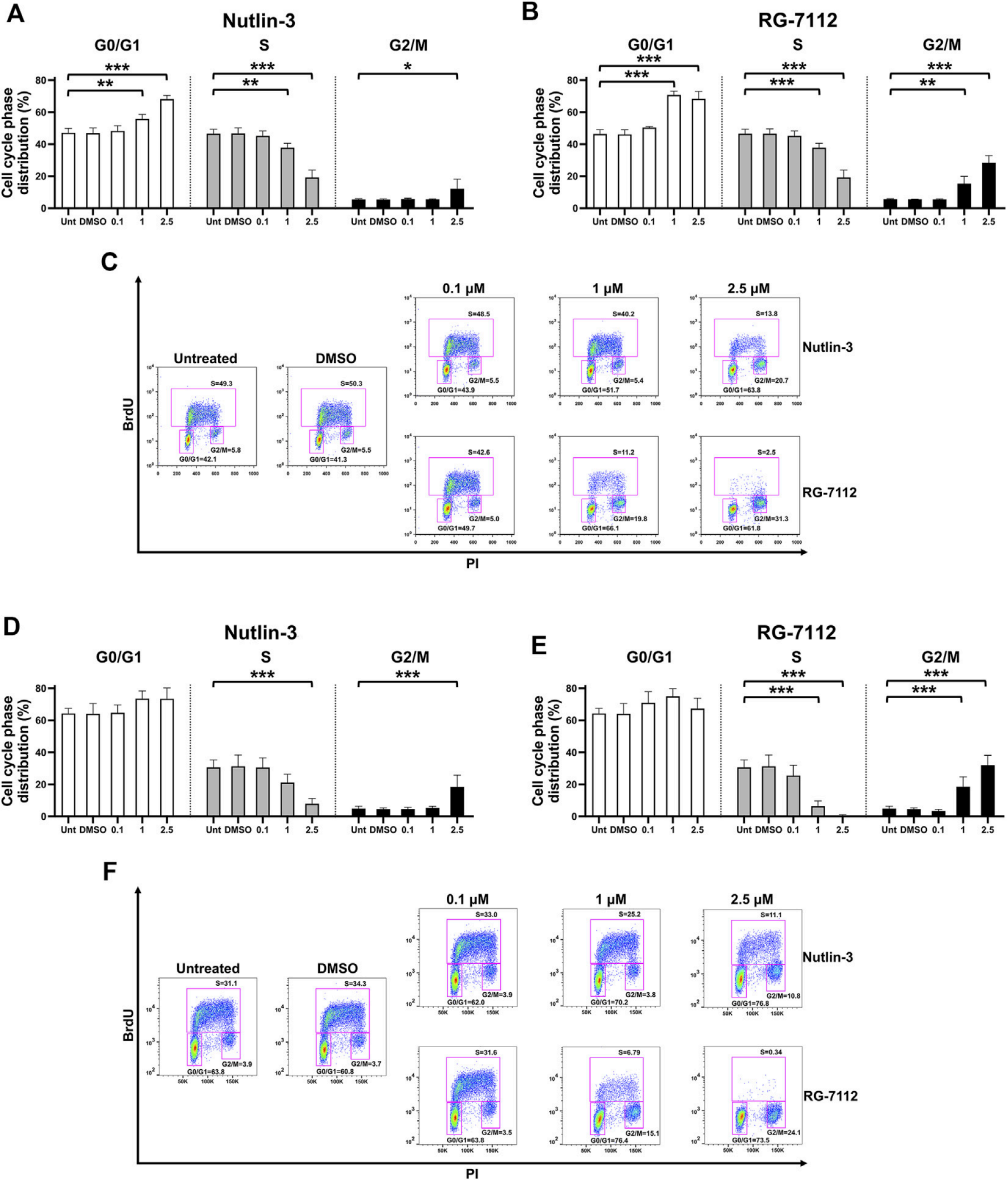
MDM2 inhibitors block cell cycle of alveolar basal epithelial cells. A549-hACE2 cells distribution in the different phases of the cell cycle calculated from cytometry dot plot deriving from BrdU/PI staining of cultures treated with different concentrations (µM) of Nutlin-3 or RG-7112 for 24 **(A–C)** and 72 h **(D–F)**. Results are expressed as percentage of total population and data are reported as mean ± standard error of the mean from three independent experiments. DMSO is reported as control vehicle. Statistical analysis was performed by ANOVA followed Bonferroni’s *post hoc* test. *p ≤ 0.05; **p ≤ 0.01; ***p ≤ 0.001. For each set of treatment, dot plot images of a representative experiment are shown **(C,F)**.

The results suggest that Nutlin-3, as well as RG-7112, significantly inhibited the cell cycle of A549-hACE2 cells in a concentration-dependent manner (Figure 1). The analyses of the cell cycle phase distribution show a significant reduction of the S phase and specular significant accumulation in G0/G1 and G2 phases, with prolonged effects until 72 h post-treatment (Figures 1A–F). In detail, compared to the untreated cultures, Nutlin-3 was able to reduce the S phase of 58.5% at the time point of 24 h (Figure 1A) and of 74.0% at 72 h (Figure 1D), when cells were treated with the higher concentration of 2.5 µM (*p* < 0.0001). RG-7112 performed even better and reduced the S phase of 71.5% and 79.2% at the time point of 24 and 72 h respectively (Figures 1B,E), at the concentration of 1 µM (*p* < 0.0001), and of 94.6% and 98.1% at the time point of 24 and 72 h respectively (Figures 1B,E) for the concentration of 2.5 µM (*p* < 0.0001), indicating an almost complete and long-lasting cell cycle block. While the significance of G0/G1 accumulation observed at 24 h (Figures 1A–C) is lost at the 72 h time point for both molecules (Figures 1D–F), the prolonged significant G2 accumulation was a typical signature indicative of the p53-p21-Rb pathway activation (Taylor et al., 2001; Engeland, 2022).

**FIGURE 2:**
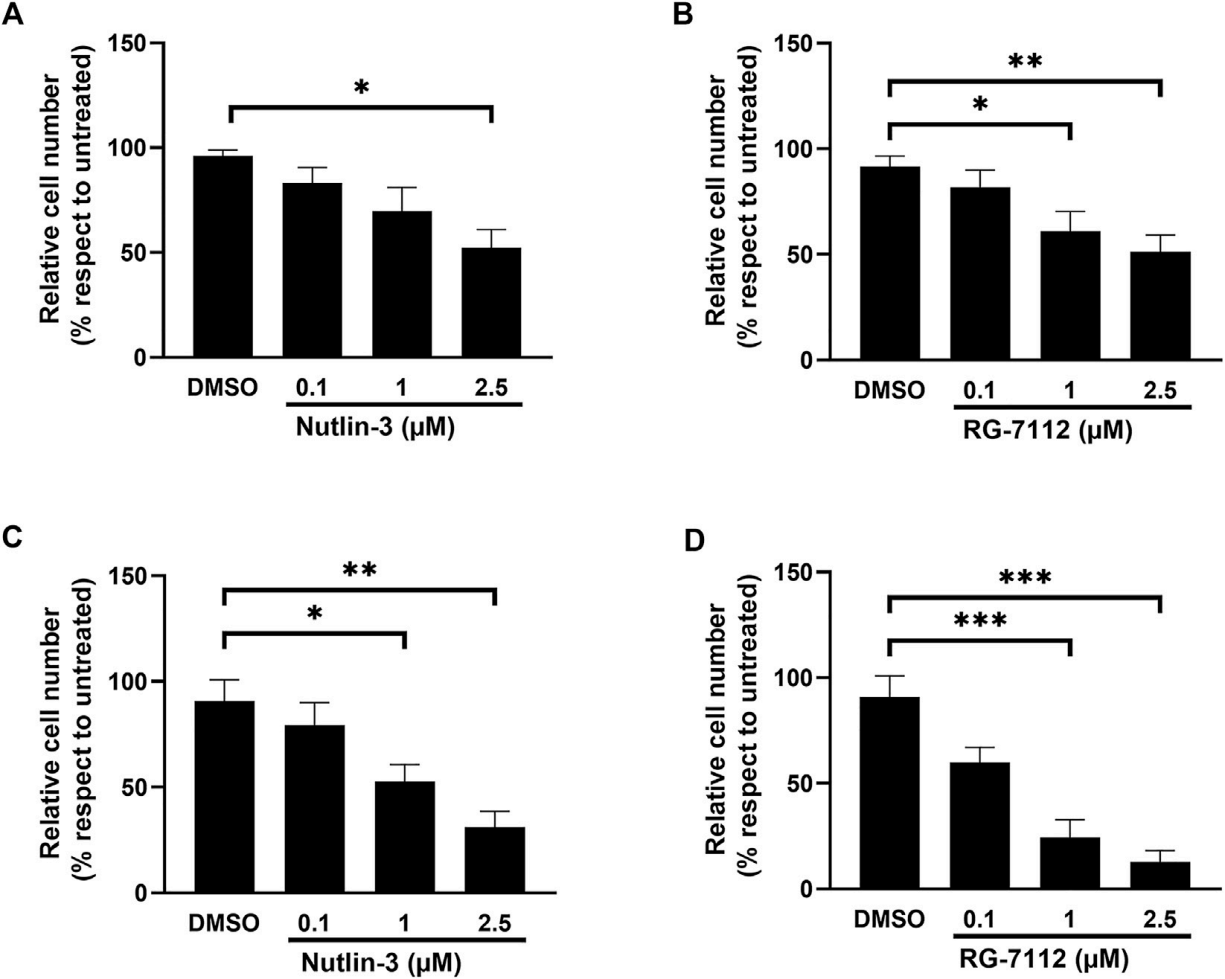
MDM2 inhibitors reduce alveolar basal epithelial cells proliferation. Cell growth analysis of A549-hACE2 cell cultures treated with different concentrations of Nutlin-3 **(A,C)** or RG-7112 **(B,D)** for 24 h **(A,B)** and 72 h **(C,D)**. Data are calculated as percentage with respect to untreated (set to 100%) and reported as mean ± standard error of the mean from three independent experiments. DMSO is reported as control vehicle. Statistical analysis was performed by ANOVA followed Bonferroni’s *post hoc* test. *p ≤ 0.05; **p ≤ 0.01; ***p ≤ 0.001.

For both drugs, the concentration-dependent cell cycle inhibition is accompanied by a significant concentration-dependent reduced cell number, due to the decreased cell replication, without significant interference with the cell viability (Figure 2 and Supplementary Figure S2). The effect on cell number was significant already at 24 h (Figures 2A,B), for Nutlin-3 at the concentration of 2.5 µM (*p* = 0.014) and for RG-7112 at the concentrations of 1 µM (*p* = 0.036) and 2.5 µM (*p* < 0.0017), and it was more pronounced at 72 h (Figures 2C,D), for Nutlin-3 at the concentrations of 1 µM (*p* = 0.0375) and 2.5 µM (*p* < 0.0057) and for RG-7112 at 1 µM (*p* = 0.0002) as well as 2.5 µM (*p* < 0.0001).

Comparing the results of the same molecular concentration at both time points, it appears evident that RG-7112 has a stronger effect on cell cycle inhibition as well on cell growth (Figures 1, 2), respect to Nutlin-3, probably due to the different affinity to MDM2.

### 3.2 The p53 pathway activated by Nutlin-3 or RG-7112 treatment of A549-hACE2 cells is maintained after SARS-CoV-2 infection

**FIGURE 3:**
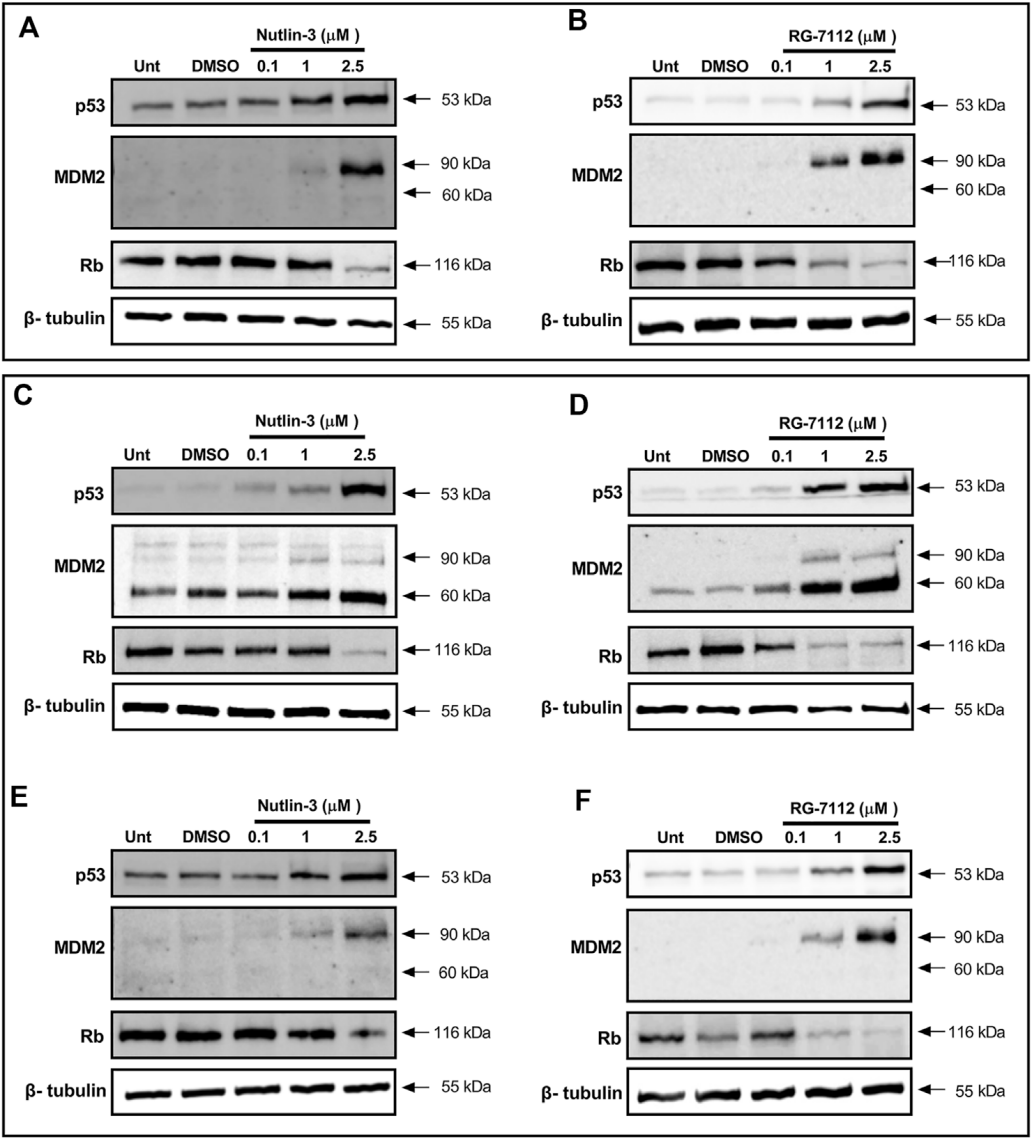
MDM2 inhibitors activate p53 pathway in alveolar basal epithelial cells. Western blotting analysis of p53, MDM2 and Rb proteins in A549-hACE2 cell cultures treated with different concentrations of Nutlin-3 **(A,C,E)** or RG-7112 **(B,D,F)** and relative controls (Unt, untreated; DMSO, control vehicle). Protein levels were evaluated 24 h after treatments **(A,B)** and 48 h after SARS-CoV-2 infection (corresponding to 72 h after initial treatment) in infected **(C,D)** and mock cultures **(E,F)**. Images are representative of three independent experiments. β-tubulin is used as internal control.

To verify the up-regulation of p53 by Nutlin-3 and RG-7112 in A549-hACE2, we evaluated by Western blotting p53 protein levels and other proteins regulated by p53, at the time of SARS-CoV-2 infection (24 h after initiation of treatment, Figures 3A,B) and 48 h post-infection (72 h of treatments, Figures 3C–F).

Results show that both molecules are able to activate p53 in a concentration-dependent manner maintaining a reliable protein level until 72 h (Figures 3A–F). Following p53 activation, results show the expected upregulation of MDM2 as a canonical p53 target gene; its induction is detectable both early (24 h, Figures 3A,B) and late (72 h, Figures 3C–F) indicating a stable concentration-dependent expression. Interestingly, only in infected cells, the 60 kDa cleaved form of MDM2 is evident and prevalent respect to the 90 kDa full-length form present in the mock-infected cells (Figures 3C–F). The p53-mediated upregulation of MDM2 seems unaffected, leading to think to a post-translational effect inducing the cleavage. Finally, to investigate the involvement of p53 in the cell cycle molecular pathway, we analyzed the trend level of retinoblastoma protein (Rb). Results confirm the BrdU analyses (Figure 1 and Supplementary Figure S3) and indicate that the cell cycle block happens through canonical Rb inhibition and that is not altered by SARS-CoV-2 infection and proliferation events (Figures 3C,D). Altogether, we observe the same mechanisms for both Nutlin-3 and RG-7112.

### 3.3 Nutlin-3 or RG-7112 treatment reduces SARS-CoV-2 release

**FIGURE 4:**
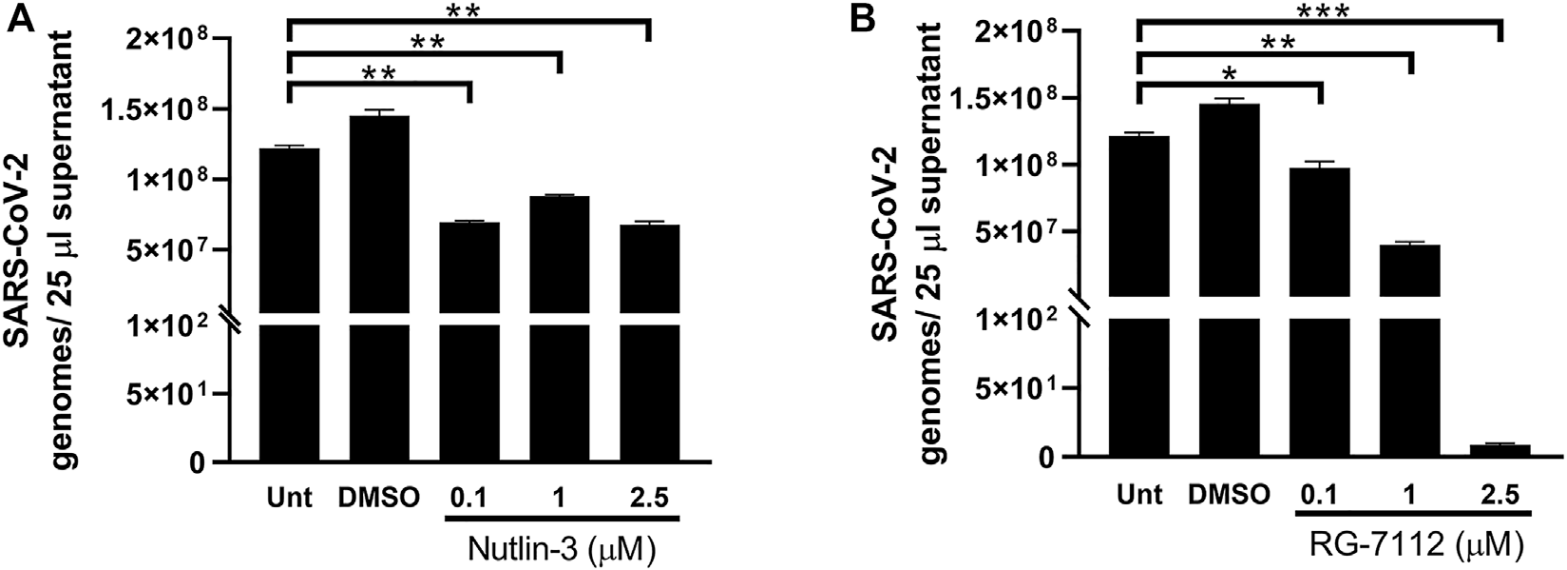
Quantification of SARS-CoV-2 infection. Quantification of SARS-CoV-2 genomes determined by RT-qPCR in 25 µL A549-hACE2 supernatants collected at 48 h post-infection of cultures treated with different concentrations of Nutlin-3 **(A)** or RG-7112 **(B)** and relative controls (Unt, untreated; DMSO, control vehicle). Results were plotted as mean ± standard error of the mean of two independent experiments run in triplicates. Statistical analysis was performed by Student’s t test. *p ≤ 0.05; **p ≤ 0.01; ***p ≤ 0.001.

In order to explore the outcome of SARS-CoV-2 infection in the presence of Nutlin-3 or RG-7112, viral RNA released from treated infected cells was next investigated. A549-hACE2 cells were treated with 0.1, one and 2.5 µM of Nutlin-3 or RG-7112 24 h prior to virus challenge to ensure the block of cell cycle at the time of infection. After 1 h of adsorption, virus inoculum was removed, and treatments were restored. The viral load was assessed 48 h post-infection (hpi). A significant reduction of SARS-CoV-2 genome copy number was detected by RT-q-PCR in the supernatants of treated cells compared to untreated cells (Figure 4). In detail, Nutlin-3 was able to affect SARS-CoV-2 replication by significantly decreasing the viral load of 43% at 0.1 µM (*p* = 0.0022); 27.7% at 1 µM (*p* = 0.0049) and 44.3% at 2.5 µM (*p* = 0.0033), in comparison to the untreated cells (Figure 4A). Regarding RG-7112, the percentages of viral copy number reduction were: 20.1% at 0.1 µM (*p* = 0.0465) 67.2% at 1 µM (*p* = 0.0016) and 99.999% at 2.5 µM (*p* = 0.0003) (Figure 4B). Moreover, the antiviral effect of RG-7112 was markedly dose-dependent and, as expected, reached higher levels compared to Nutlin-3, most likely due to its enhanced MDM2-binding affinity.

### 3.4 Nutlin-3 or RG-7112 treatment down-regulates the pro-inflammatory status induced by virus infection

**FIGURE 5:**
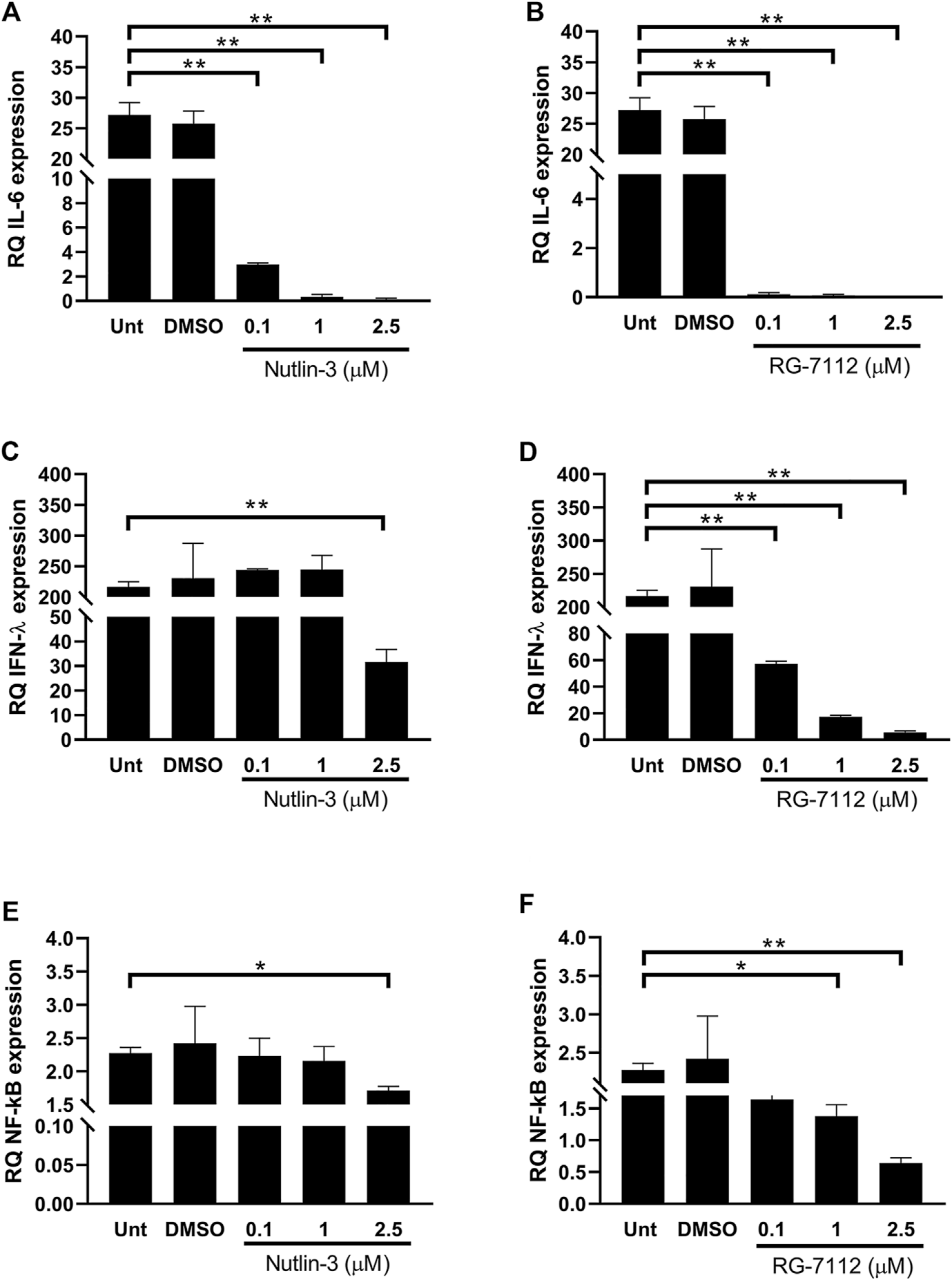
Quantification of inflammatory environment. Relative quantification (RQ) of expression levels of IL-6 **(A,B)** and IFN-λ1 **(C,D)** and NF-κB **(E,F)**, in SARS-CoV-2-infected A549-hACE2 cells treated with different concentrations of Nutlin-3 **(A,C,E)** or RG-7112 **(B,D,F)** and relative controls (Unt, untreated; DMSO, control vehicle). Gene expression was normalized to GAPDH housekeeping gene, and quantification is relative to not infected untreated cells. Mean ± standard error of the mean of two independent experiments in triplicate are shown. Statistical analysis was performed by Student’s t test. *p ≤ 0.05; **p ≤ 0.01.

The immune response to virus infections involves a robust innate anti-viral response meditated by type I and III interferons (IFNs) and a potent inflammatory response enhancing both rapid immune cell recruitment and damage of infected cells and tissues. IL-6 is a multi-tasking cytokine with high activity in patients with severe SARS-CoV-2 infection (Majidpoor and Mortezaee, 2022). IFN-λ acts in specific tissues and it functions mainly at the epithelial barrier surfaces (Lazear et al., 2015). The treatment with both Nutlin-3 and RG-7112 triggered to a huge decrease of IL-6 expression in infected cells (Figures 5A,B), with significant fold differences at all the tested concentrations respect to control. After SARS-CoV-2 infection, IFN- λ expression is greatly up-regulated when compared to uninfected cells (Figures 5C,D). The administration of 2.5 µM Nutlin-3 was able to significantly reduce the expression of IFN-λ (*p* = 0.0027) and, more relevant, RG-7112 treatment down-regulated IFN-λ in a dose dependent manner, with an almost complete loss of its expression (Figures 5C,D).

The expression of both IL-6 and IFN-λ are controlled by NF-κB transcription factor, that is implicated in expression of type I and type III IFNs and pro-inflammatory cytokines (Bartlett et al., 2012). The administration of Nutlin-3 and RG-7112 in SARS-CoV-2-infected cells was able to reduce the expression levels of NF-κB compared to untreated infected cells (Figures 5E,F). The decrease due to Nutlin-3 treatment reached significant values when used at 2.5 µM (*p* = 0.034), whereas RG-7112 treatment showed a dose-dependent trend of down-regulation, with significant reductions at one and 2.5 µM concentrations (*p* = 0.0458 and *p* = 0.0054, respectively).

## 4 Discussion

Virtually, all viruses target molecular cellular mechanisms that can control their replication, dissemination, or persistence. Among these is p53, a key gatekeeper for cell division and survival that also regulates innate immune responses (Alzhanova et al., 2021). The expression and activity of key transcriptional factors, such as p53 and NF-kB are altered upon SARS-CoV-2 infection, as well as other key molecules such as the virus host cell entry mediator ACE2, member of the renin-angiotensin system RAS-pathway. These modifications might play a central role in the impaired immune response and in the cytokine release during severe form of SARS-CoV-2 infection. The S2 subunit of SARS-CoV-2 strongly interact with p53 (Singh and Bharara Singh, 2020), that in turn can regulate ACE2 receptor in a tissue- and sex-specific fashion (Zhang et al., 2021). Moreover, the viral SARS-unique domain, SUD, and papain-like protease, PLpro, stabilize RCHY1 who mediates p53 degradation (Ma-Lauer et al., 2016). PLpro stabilizes MDM2 itself leading in turn to accelerated proteasome degradation of basal p53 (Dong et al., 2020).

The aim of our work was to evaluate a new approach against the virus, by using MDM2 inhibitors to effectively raise p53 levels and activate p53-dependent pathways including cell cycle inhibition. Indeed, so far, it was unknown the capacity of the virus to proliferate under these conditions. We used two MDM2 inhibitors (Nutlin-3 and RG-7112) to perform a proof-of-concept evaluation, observing that both drugs are effective in inducing cell cycle block and in reducing SARS-CoV-2 replication in A549-ACE2 cells. In addition to the direct effect on viral replication, these treatments are effective in modulating cytokines expression during SARS-CoV-2 infection. It has been clearly shown that SARS-CoV-2 induces an inflammatory response that involves the activation of NF-kB, a key element in the well described “cytokine storm” observed in patients affected by SARS-CoV-2 (Sallenave and Guillot, 2020). In particular, SARS-CoV-2 nucleocapsid protein (N) mediated IL-6 expression occurs *via* the direct binding of N protein on NF-κB promoter and *via* facilitation of the nuclear translocation of NF-κB (Zhang et al., 2007). We observed that both Nutlin-3 and RG-7112 triggered to a huge decrease of IL-6 expression in infected cells, with significant fold differences at every tested concentrations. Coherently, the administration of Nutlin-3 and RG-7112 to SARS-CoV-2-infected cells was able to reduce the expression levels of NF-κB compared to untreated infected cells. NF-κB clearly plays a critical role in the signal transduction pathway that senses viral nucleic acids during pathogenic infection (Pfeffer et al., 2011). In particular, SARS-CoV-2 induces toll like receptor-7, TLR7, pathway *via* NF-κB, inducing type 1 IFN, IFN-γ, and IFN-λ3, starting from 48 h post-infection (Bortolotti et al., 2021; Caccuri et al., 2022). The lambda interferons constitute the most recently discovered IFN family with anti-viral activity (Swider et al., 2014) and their expression is controlled by NF-kB. After SARS-CoV-2 infection, expression of IFN- λ is greatly up-regulated when compared to uninfected cells. In our setting, the administration of Nutlin-3 and RG-7112 was able to significantly reduce the expression of IFN-λ to very low level. These results are in line with the reduced replication of SARS-CoV-2 in Nutlin-3 and RG-7112 treated cells, that sense lower levels of viral nucleic acids and reduced the expression of IFNs genes.

Since our study was done using a stabilized alveolar basal epithelial cell line, some concerns could be related to the fact that A549-hACE2 are not normal cells. At present, it is not known how a non-cancer SARS-CoV-2-infected cell could respond to MDM2 inhibitors, but their use has been recently proposed as a possible strategy for COVID-19 patients (Zauli et al., 2020; Zauli et al., 2022). As in cancer, also in SARS-Cov-2 infected cells p53 is kept under control by altered MDM2 levels, as direct or indirect virus-mediated effect (Yuan et al., 2015; Ma-Lauer et al., 2016; Cardozo and Hainaut, 2021; Kumar et al., 2022). We believe that the A549-hACE2 is an appropriate model for the present study, considering also that we did not see drug-related cytotoxicity. Non-etheless, we are aware that other insights, such as the research of further models closer to natural infection, are needed before translation to *in vivo* studies.

Moreover, further reasoning could be related to the efficacy of MDM2 inhibitors in normal cells. It was observed that MDM2 inhibitors are not cytotoxic in normal healthy PBMCs and in primary human fibroblasts (Carvajal et al., 2005; Voltan et al., 2014). Moreover, it was observed a reversible cell-cycle arrest in primary fibroblasts, in primary epithelial cells and in endothelial cells (van Leeuwen et al., 2012; Huang et al., 2009; Secchiero et al., 2007) and new preclinical data are emerging about effects on aged epidermal cells and adipose tissue cells (Charruyer et al., 2021; Zhao et al., 2022). These observations can open to new therapeutic opportunities, but at the same time indicate that further preclinical and clinical investigations on MDM2 inhibitors need to be done. In this frame, we believe that RG-7112 or other MDM2-inhibitors with higher bioavailability than Nutlin-3 will be plausible and appropriate for future research involving *in vivo* models for SARS-CoV-2 study.

## 5 Conclusion

Our results confirmed the literature data, showing a decrease of p53 and an increase of MDM2 levels during SARS-CoV-2 infection. It is interesting to underline that SARS-CoV-2 infections induced a peculiar form of MDM2, the 60kDa, that is clearly evident only during SARS-CoV-2 infection, and that was enhanced following Nutlin-3 and RG-7112 treatments. MDM2 can be cleaved by caspase 3 (CPP32) after aspartic acid-361, generating a 60 kDa fragment (Pochampally et al., 1998). In addition, also caspase two have been recently described as the biological scissors of MDM2 and shown to have non-apoptotic function in regulating cell division (Boice et al., 2022). Importantly, caspase two and cleaved 60 kDa MDM2 are involved in a positive feedback loop of p53 auto-control, that induces stabilization of p53 (Oliver et al., 2011). Apparently, p53 pathways activated after MDM2 inhibitors treatment didn’t change after SARS-CoV-2 infection and, so far, we can only hypothesize that the virus could directly or indirectly mediate the cleavage of MDM2.

Interestingly, p53 and NF-kB balance is critically altered upon SARS-CoV-2 infection, and the shift towards NF-kB might affect the immune response and aberrant cytokine release of the disease (Milani et al., 2022). To take control above NF-kB, the upregulation of p53 levels by MDM2 inhibitors can be the key for lowering the inflammation status of the severe form of the infection. Using Nutlin-3 and RG-7112 as a tool, we demonstrated that p53 can “extinguish the fire” induced upon NF-kB expression under SARS-CoV-2 infection.

Further confirmations of our results will postulate alternative therapies to counteract the molecular mechanisms by which SARS-CoV-2 modulates p53 and NF-kB expression and activity in order to maximize viral replication into the host cells. To this end, the repositioning of existing drugs as well as the development of new active compounds may be of great relevance in the view of synergic treatments able from one side to improve the immune status of individuals and on the other side to target specific pathways involved in disease development and progression.

## Data Availability

The original contributions presented in the study are included in the article/Supplementary Material, further inquiries can be directed to the corresponding authors.
