# Supplementary material for: Cell cycle block by p53 activation reduces SARS-CoV-2 release in infected alveolar basal epithelial A549-hACE2 cells
Source: Front Pharmacol. 2022 Dec 13;13:1018761. doi: 10.3389/fphar.2022.1018761 (PMC9792496; doi:10.3389/fphar.2022.1018761)
Supplement: Supplementary file 1 [file DataSheet2.PDF]

Supplementary Material

1 Supplementary Figures

Supplementary Figure 1

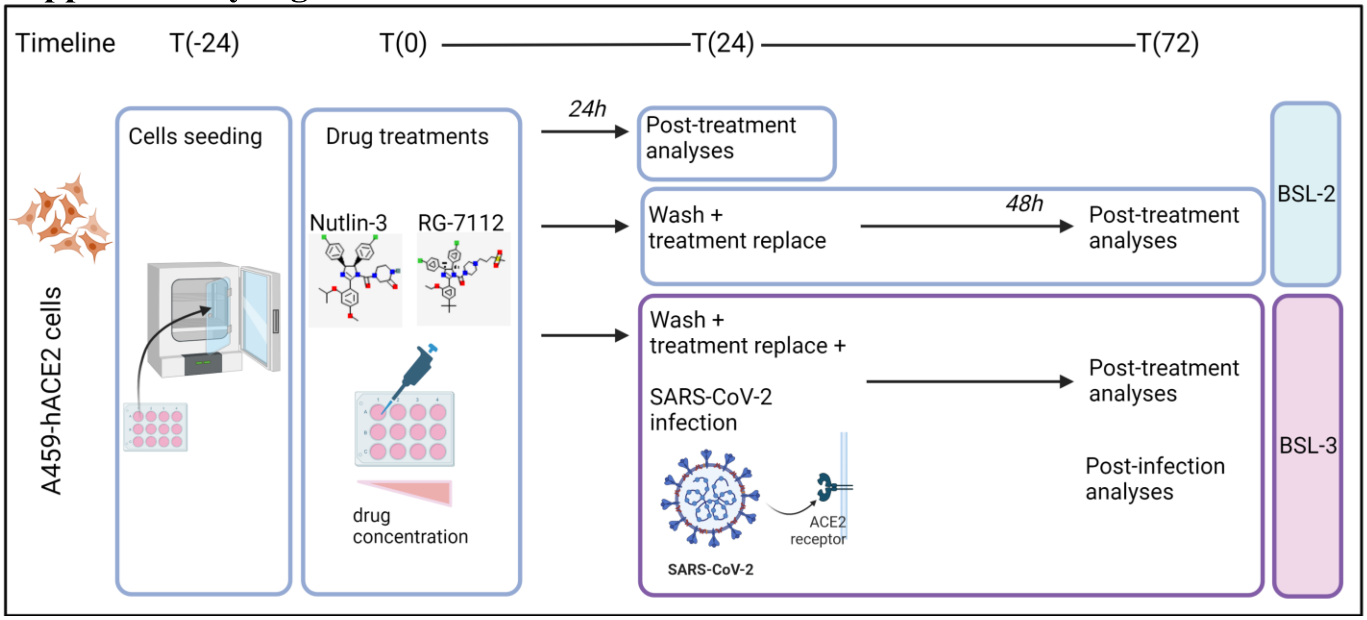

Supplementary Figure 1. Experimental workflow. The day before the experiments (T-24), cells were seeded at a density of 8x10^{4} cells/mL/well in 12 well plates. The following day (T0), cells were treated with 0.1, 1 and 2.5 µM of Nutlin-3 or RG-7112 or left with complete medium (Untreated) or treated with DMSO as drug vehicle (DMSO). After 24 hours of treatment (T24), cells were assayed for cell cycle and cell growth, apoptosis and western blotting analyses. Parallel cultures were washed and left for 48 hours in complete medium supplemented with 5% fetal bovine serum with treatment replacement. At (T72) cells were harvested for analyses in BLS-2 laboratory. At (T24), some cultures were infected in BLS-3 laboratory. Cells were washed and treatments were restored. After 48 hours (T72) samples were collected for post-infection analysis. Created with BioRender.com.

Supplementary Figure 2

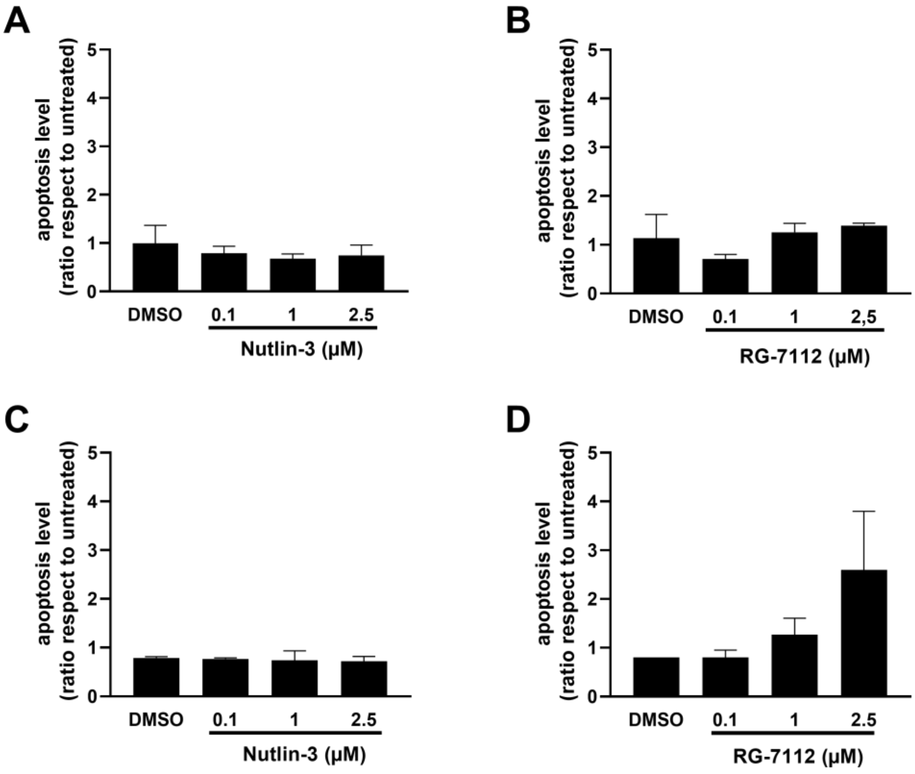

Supplementary Figure 2. MDM2 inhibitors reduce alveolar basal epithelial cells proliferation without inducing apoptosis. Apoptosis analysis of A549-hACE2 cell cultures treated with different concentrations of Nutlin-3 or RG-7112 for (A-B) 24 and (C-D) 72 hours. Data are calculated from hypodiploid percentage values after BrdU/PI staining and reported as mean ± standard error of the mean from three independent experiments. DMSO is reported as vehicle-control. Statistical analysis was performed by ANOVA followed by Bonferroni's post hoc test.

Supplementary Figure 3

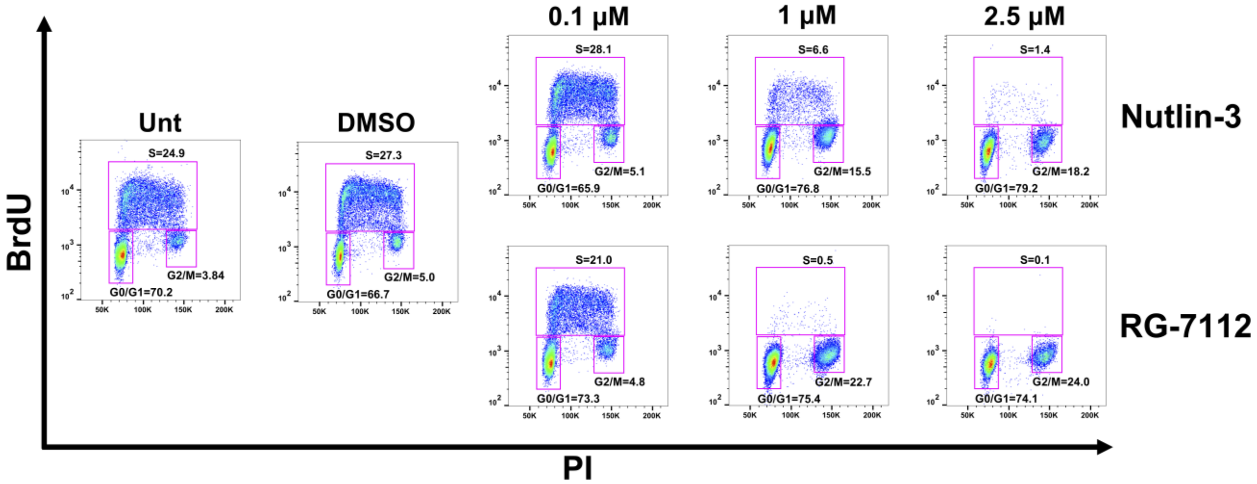

Supplementary Figure 3: MDM2 inhibitors induce cell cycle block after SARS-CoV-2 infection of alveolar basal epithelial cells. A549-hACE2 cells distribution in the different phases of the cell cycle following BrdU/PI staining of cultures treated with different concentrations of Nutlin-3 or RG-7112 for 48 hours after SARS-CoV-2 infection at a MOI of 0.01. DMSO is reported as control vehicle. Results are expressed as percentage of total population. Dot plot images of a representative experiment are shown.
