# Supplementary figures and images for: Cell cycle block by p53 activation reduces SARS-CoV-2 release in infected alveolar basal epithelial A549-hACE2 cells
Source: Front Pharmacol. 2022 Dec 13;13:1018761. doi: 10.3389/fphar.2022.1018761 (PMC9792496; doi:10.3389/fphar.2022.1018761)

## Raw Data

Row images from the Western blotting experiments presented in **Figure 3**.

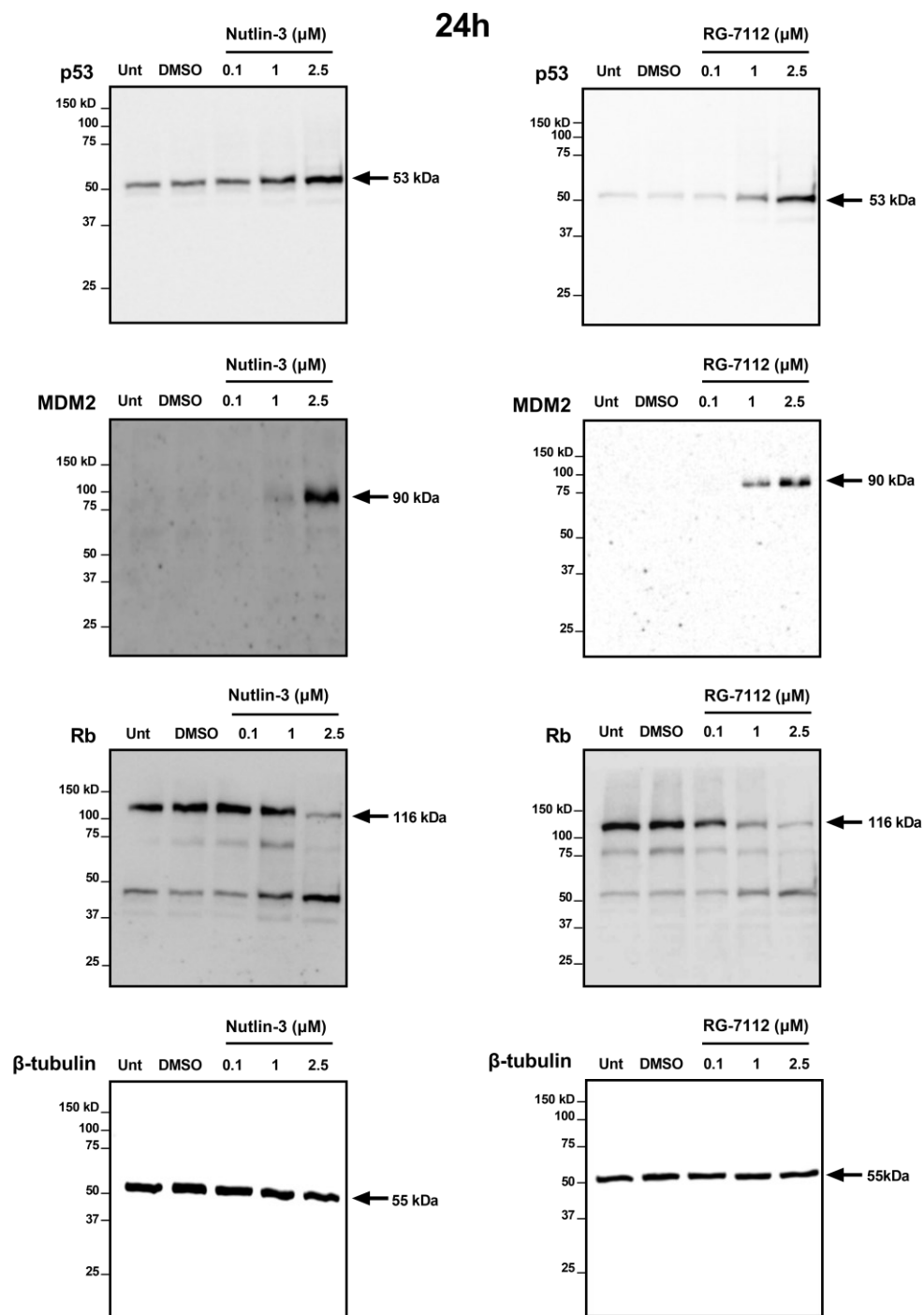

## SARS-CoV-2

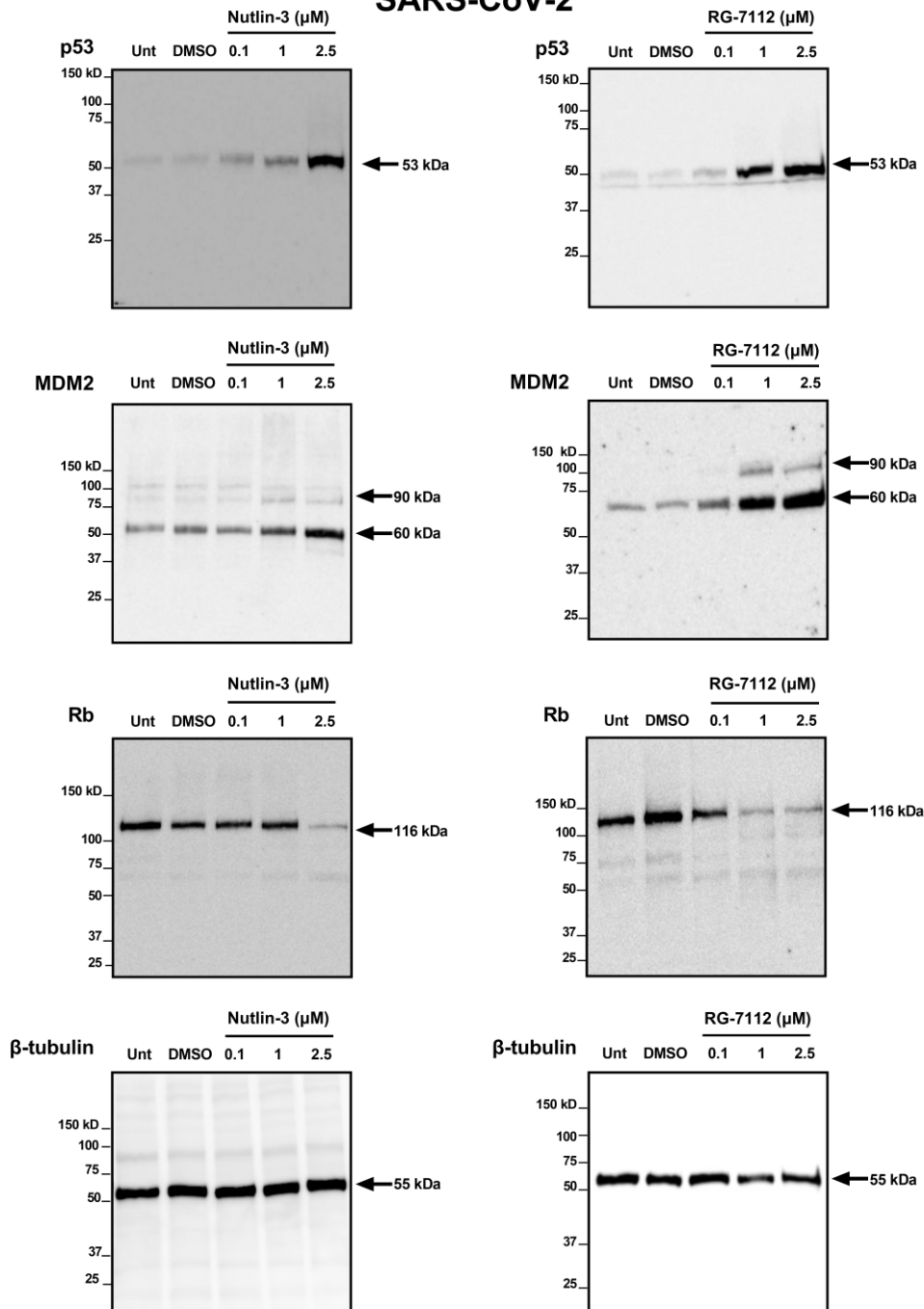

# Mock

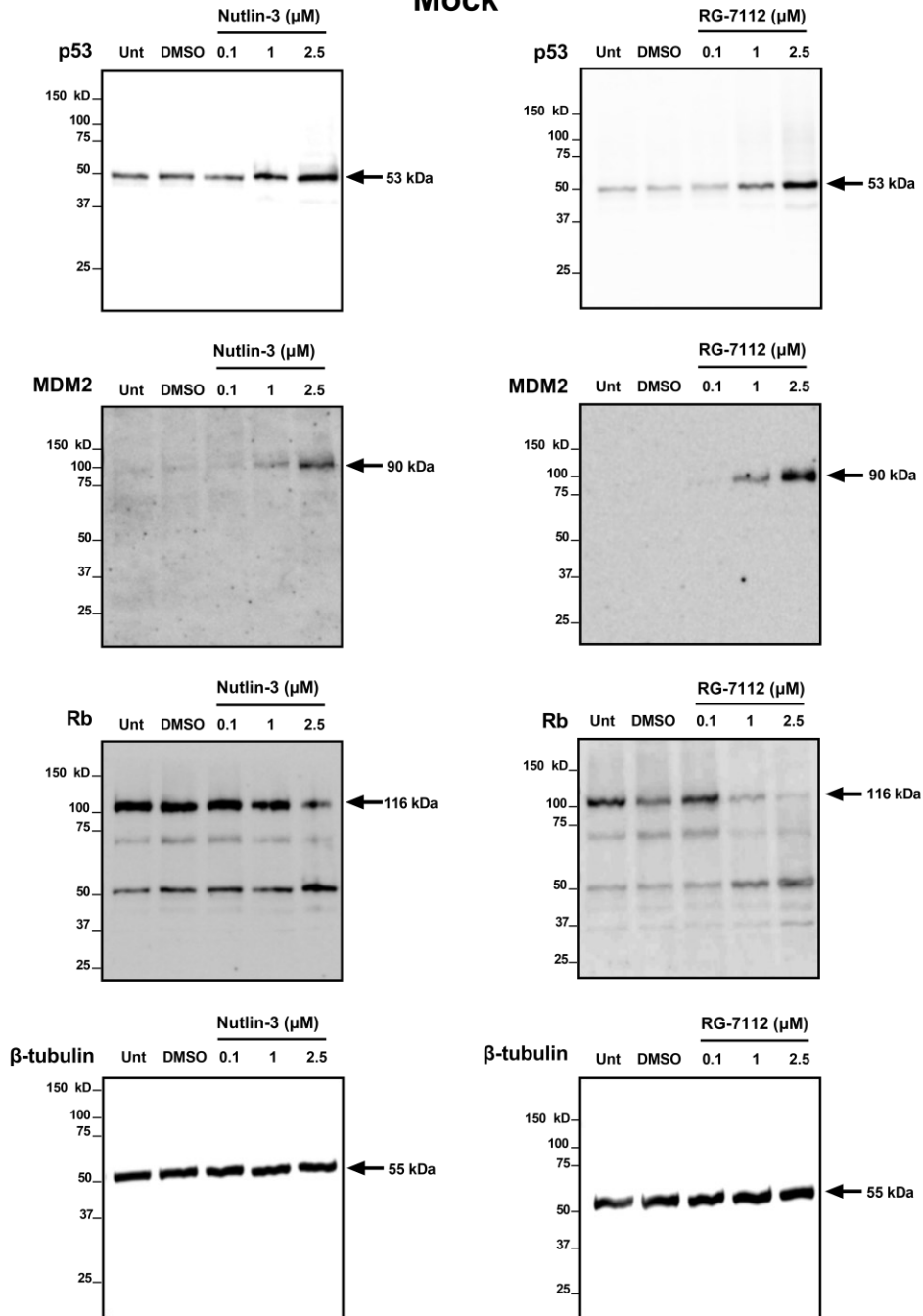

Supplement: Supplementary file 2 [file DataSheet1.PDF]
